# Developing a kinematic understanding of chest compressions: the impact of depth and release time on blood flow during cardiopulmonary resuscitation

**DOI:** 10.1186/s12938-015-0095-4

**Published:** 2015-11-04

**Authors:** Joshua W. Lampe, Yin Tai, George Bratinov, Theodore R. Weiland, Christopher L. Kaufman, Robert A. Berg, Lance B. Becker

**Affiliations:** The Center for Resuscitation Science, Department of Emergency Medicine, University of Pennsylvania, 3501 Civic Center Blvd, Suite 6026, Philadelphia, PA 19104 USA; Department of Anesthesiology and Critical Care Medicine, The Children’s Hospital of Philadelphia, Philadelphia, PA USA; ZOLL Medical Corporation, Chelmsford, MA USA

**Keywords:** Resuscitation, Cardiopulmonary resuscitation, Chest compressions, Blood flow

## Abstract

**Background:**

Effective cardiopulmonary resuscitation is a critical component of the pre-hospital treatment of cardiac arrest victims. Mechanical chest compression (MCC) devices enable the delivery of MCC waveforms that could not be delivered effectively by hand. While chest compression generated blood flow has been studied for more than 50 years, the relation between sternum kinematics (depth over time) and the resulting blood flow have not been well described. Using a five parameter MCC model, we studied the effect of MCC depth, MCC release time, and their interaction on MCC generated blood flow in a highly instrumented swine model of cardiac arrest.

**Methods:**

MCC hemodynamics were studied in 17 domestic swine (~30 kg) using multiple extra-vascular flow probes and standard physiological monitoring. After 10 min of untreated ventricular fibrillation, mechanical MCC were started. MCC varied such that sternal release occurred over 100, 200, or 300 ms. MCC were delivered at a rate of 100 per min and at a depth of 1.25″ (n = 9) or at a depth of 1.9″ (n = 8) for a total of 18 min. Transitions between release times occurred every 2 min and were randomized. Linear Mixed Models were used to estimate the effect of MCC depth, MCC release time, and the interaction between MCC depth and release time on physiological outcomes.

**Results:**

Blood pressures were optimized by a 200 ms release. End tidal carbon dioxide (EtCO_2_) was optimized by a 100 ms release. Blood flows were significantly lower at a 300 ms release than at either a 100 or 200 ms release (*p* < 0.05). 1.9″ deep MCC improved EtCO_2_, right atrial pressure, coronary perfusion pressure, inferior vena cava blood flow, carotid blood flow, and renal vein blood flow relative to 1.25″ MCC.

**Conclusions:**

Deeper MCC improved several hemodynamic parameters. Chest compressions with a 300 ms release time generated less blood flow than chest compressions with faster release times. MCC release time is an important quantitative metric of MCC quality and, if optimized, could improve MCC generated blood flows and pressures.

**Electronic supplementary material:**

The online version of this article (doi:10.1186/s12938-015-0095-4) contains supplementary material, which is available to authorized users.

## Background

When chest compression was first described it was believed that the heart was directly compressed between the sternum and the spine (the cardiac pump model), emptying the ventricles, generating forward flow [1]. Two decades later, the thoracic pump model was proposed wherein the chest compression increases the intra-thoracic pressure, expelling blood from the heart and the blood vessels in the chest with extra-thoracic venous valves enforcing physiologically forward directionality on the resulting blood flow [2–4]. While these models enable mechanistic interpretations of chest compression generated blood flow, the connection between sternum kinematics (position, velocity, acceleration, and time) and intra-thoracic pressure or ventricular compression remains poorly described.

This disconnect is important because clinical descriptions of CPR quality are given in terms of sternum kinematics. In 2010, the International Liaison Committee on Resuscitation published resuscitation guidelines that described “quality” chest compressions as compressions at a depth greater or equal to 2″, at a rate of at least 100 compressions per minute (cpm) while ensuring complete chest compression release [5–7]. This description is clinically pragmatic, reflecting what is practicable for delivery of manual chest compressions and what is measureable. While it would make more mechanistic sense to compress to a desired intra-thoracic pressure or percent of ventricular compression, these measurements are practically impossible to make during a resuscitation. Finally, the advent of mechanical chest compression devices, which make it possible to prescribe sternum kinematics with a high degree of precision and accuracy, requires a kinematic understanding of chest compression generated blood flow to enable the development of optimal chest compression waveforms.

There are multiple obstacles to relating chest compression hemodynamics to sternum kinematics. Currently, sternum kinematics are measured by accelerometer [8]. Integrating acceleration signals to estimate velocity and position is known to be problematic, and significant signal processing and a priori signal correction are required to provide a reasonable estimate. In addition, the current kinematic description of CPR is only complete if it is describing a sine wave, which does not accurately describe manual or mechanical compressions [9]. As a result, there are an infinite number of chest compression waveforms that fit the quality chest compression description [10, 11]. There are also several issues from a hemodynamic measurement perspective which contributes to the problem. Systemic blood flow has been measured with radioactive or fluorescent microspheres as tracer particles in the blood [3, 12–14]. This technique estimates time averaged blood flow by counting the number of tracer particles present in post-mortem tissue samples. It is difficult to use this technique to study the effect of chest compression kinematics because the data is time averaged over several minutes. Other studies use trans-vascular Doppler ultrasound measurement of blood flow in a single vessel, usually the common carotid artery [9, 15, 16]. While this measurement provides significantly better time resolution, data from a single blood vessel is not sufficient to relate sternum kinematics to chest compression generated blood flow.

In this manuscript we report blood flows from a novel high-fidelity swine model in which we studied the impact of changes in sternum kinematics, via changes in chest compression depth and chest compression release time, on blood flow generated in six blood vessels with perivascular Doppler ultrasound measurements during ongoing cardiac arrest. Our interest in chest compression release time is related to recent reports on the negative impact of leaning on CPR outcomes [17–20] and conflicting clinical data relating chest compression release velocity to survival [10, 11].

## Methods

The study was approved by the Institutional Animal Care and Use Committee of the University of Pennsylvania and the Children’s Hospital of Philadelphia. All animals received treatment and care in compliance with the 1996 Guide for the Care and Use of Laboratory Animals by the National Research Council in accord with the USDA Animal Welfare Act, PHS Policy, and the American Association for Accreditation of Laboratory Animal Care. All studies were conducted by qualified personnel.

### Animal preparation

Seventeen domestic swine (30.5 ± 1.68 kg) were sedated with intramuscular ketamine (20 mg kg^−1^) and xylazine (2 mg kg^−1^), followed by induction of general anaesthesia by mask administration of 4 % isoflurane in 100 % oxygen. After endotracheal intubation, a surgical plane of anaesthesia was maintained on isoflurane and a mixture of air and oxygen, adjusted to achieve an inspiratory oxygen fraction of 0.4. The animals were mechanically ventilated with a pressure controlled ventilator (Modulus SE 7900; Datex-Ohmeda Inc., USA) with a tidal volume of 12 mL/kg, PEEP 6 cm H_2_O, and rate of 12 breaths/min. The rate was titrated to maintain end-tidal carbon dioxide (EtCO_2_) at 38–42 mmHg (NICO_2_, Novametrix Medical Systems Inc.).

After aseptic preparation, solid state pressure transducers (MPC-500, Millar Instruments) were advanced through introducers in the right femoral artery and vein to measure the aortic pressure (AOP) and the right atrial pressure (RAP) respectively.

After aseptic preparation, a surgical cutdown procedure was used to expose the right common carotid artery and the right external jugular vein. After surgical exposure, ultrasound blood flow probes were placed around the vessels (PS-3, Transonic Systems Inc, USA) and secured with sutures. After a laparotomy, ultrasound flow probes were placed around the right renal artery and vein, the inferior vena cava, and the abdominal aorta (Renal vessels: PS-2.5, Aorta: PAU-10, IVC: PAU-12, Transonic Systems Inc, USA). The incisions in the neck and the abdomen were sutured shut after sensor placement.

If the mean arterial pressure was below 60 mmHg, a 20 ml/kg infusion of saline was provided at the end of the surgical prep period, in an attempt to offset fluid loss due to fasting and third space losses during surgical preparation. Self adhesive hook and loop fasteners were attached to the chest compressor and the sternum of the animal. This ensured that the sternum and the compressor head were in constant contact during chest compressions and therefore ensured the release time of the chest was the same as the compressor head.

After recording baseline values, ventricular fibrillation (VF) was electrically induced and the ventilator was turned off. After 10 min of untreated VF, chest compressions were initiated as described below. During the CPR period, ventilations were provided at the tidal volume used before cardiac arrest at a rate of 6 ventilations per minute with 100 % FiO_2_ and no PEEP.

Chest compressions were controlled by a computer, which defined a chest compression based on five parameters: depth, compression time, compression hold time, release time, and release hold time, as shown in Additional file 1: Figure S1. Chest compressions were provided at a rate of 100 per min. Chest compressions were provided at a depth of 1.25″ (n = 9) and 1.9″ (n = 8). These two depths were chosen to represent the upper and lower bounds of guideline chest compression depths as defined by the 2005 and 2010 CPR guidelines. The motor driving the piston could not complete a 2.0″ compression in 100 ms so we used 1.9″. Three chest compression waveforms were used. For all waveforms (WF), the compression time was held constant at 100 ms and the compression hold time was held constant at 200 ms. WF 1 used a release time of 100 ms and a release hold time of 200 ms. WF 2 used a release time of 200 ms and a release hold time of 100 ms. WF 3 used a release time of 300 ms and a release hold time of 0 ms. Each WF was performed for a 2 min epoch. Animals were randomized to three groups where the chest compression WF followed the patterns outlined in Table 1.

**Table 1:** Chest compression waveform patterns for the three experimental groups

Group	Epoch								
	1	2	3	4	5	6	7	8	9
1	WF 1	WF 2	WF 3	WF 2	WF 3	WF 1	WF 3	WF 1	WF 2
2	WF 2	WF 3	WF 1	WF 3	WF 1	WF 2	WF 1	WF 2	WF 3
3	WF 3	WF 1	WF 2	WF 1	WF 2	WF 3	WF 2	WF 3	WF 1

Each pattern was repeated three times

### Data analysis

**Fig. 1:**
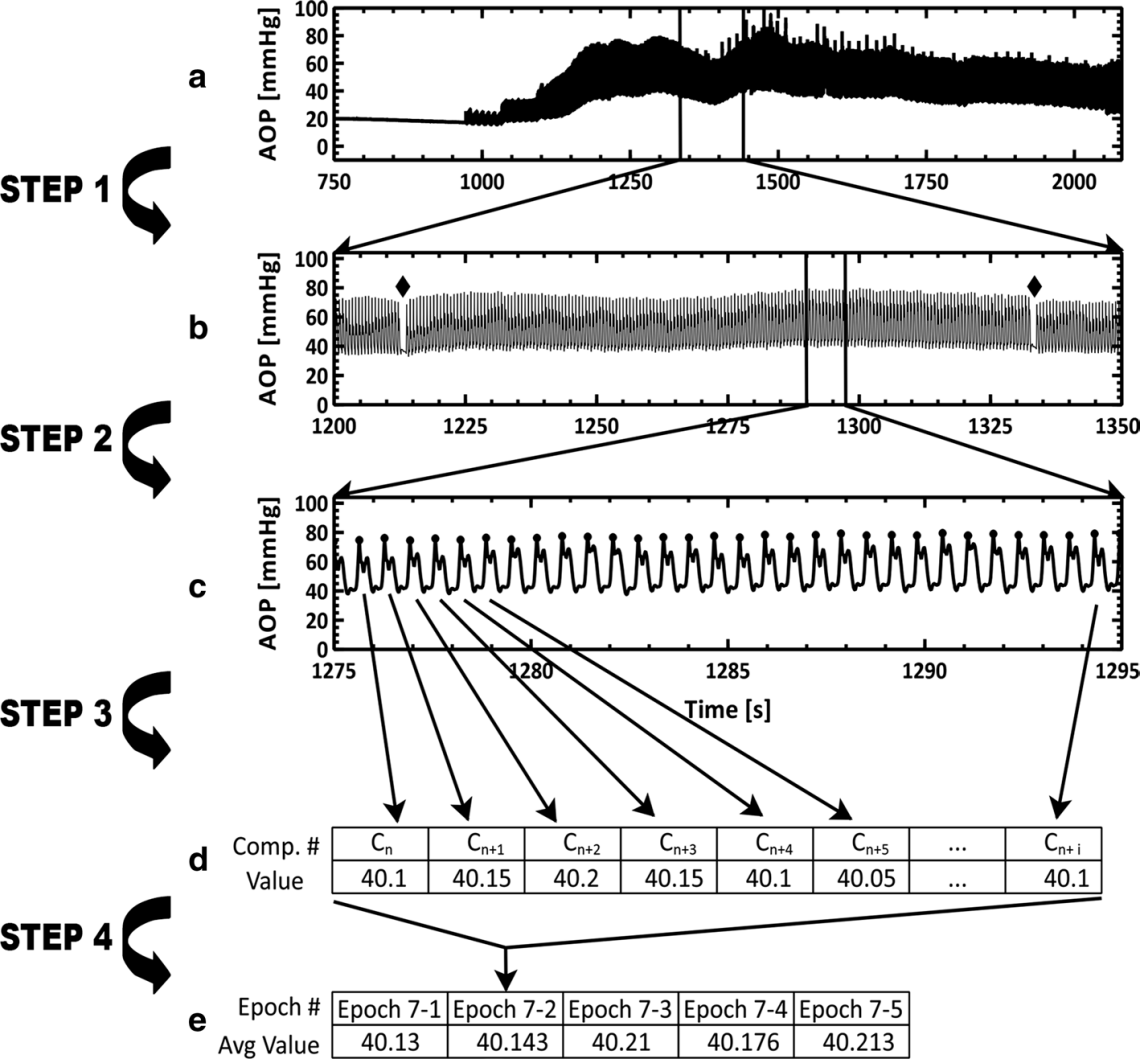
Schematic of the data analysis process. The process is illustrated here using aortic pressure data, but this process was applied to all data streams. Briefly, at the initiation of analysis the locations of the transitions from one chest compression waveform to another were determined (*Step 1*) and the locations of the chest compression maxima were determined (*Step 2*). Once the waveform transitions and chest compression locations were determined, those locations were used to segment data into values per chest compression (*Step 3*) and five average values (approximately 20 s of data) per epoch (*Step 4*). *Step 1* and *Step 2* were typically completed on the aortic pressure or the venous pressure data channel. *Steps 3* and *4* were completed on all data channels

The top graph in Fig. 1 shows the aortic pressure during untreated VF and 18 min of CPR. In Step 1, the transition between chest compression waveforms are located. The waveform transitions are found using a purpose built computer script and the results are inspected and verified/corrected manually, resulting in the location of waveform transitions, indicated by diamonds in the second graph in Fig. 1.

During Step 2, within each CPR epoch, each complete chest compression is identified, as shown in the third graph in Fig. 1. During Step 3, depending on the calculation, the following analyses are performed: averaging, integrating, location of local maxima and minima, location of decompression. Each value is recorded in an array for the epoch as shown in the first table in Fig. 1. In Step 4, each array was divided into fifths, and the values were averaged for each fifth of the epoch, as shown in the second table in Fig. 1.

### Statistical analysis

Four statistical models were used to explore the possible relationships between chest compression release time and depth and the measured hemodynamics. The first and second models are univariate models exploring the impact of chest compression release time (model 1) and chest compression depth (model 2). The third model is a covariate model that explores the impact of both chest compression release time and chest compression depth (model 3). Statistical outcomes from models 1, 2, and 3 are shown in Table 2. The fourth model is a multivariate model that explores the impact of chest compression release time, chest compression depth, and the linear interaction between depth and release time (model 4). Statistical outcomes from model 4 are shown in Table 3. Both release time and depth were considered as class variables and the references classes are WF = WF 3 and Depth = 1.25″.

**Table 2:** Statistical dependence of physiological measures on changes in chest compression waveform or depth

Measurement	Model 1: WF analysis	Model 2: Depth analysis	Model 3: WF + Depth analysis	
	WF	Depth	WF	Depth
AOP	*WF 2* *>* *WF 1, WF 3*	ns	*WF 2* *>* *WF 1, W F3*	ns
RAP	*WF2* *>* *WF 1*	*1.25″* *>* *1.9″*	ns	*1.25″* *>* *1.9″*
CPP	*WF 2* *>* *WF 3;* WF 2 > WF 1	*1.9″* *>* *1.25*″	*WF 2* *>* *WF1, WF 3*	*1.9″* *>* *1.25″*
EtCO_2_	WF 1 > WF 2	*1.9″* *>* *1.25″*	ns	*1.9″* *>* *1.25″*
AOR flow	WF 3 < WF 1,WF 2	ns	WF 3 < WF 1, WF 2	ns
IVC flow	ns	*1.9″* *>* *1.25″*	ns	*1.9″* *>* *1.25″*
CAR flow	*WF 3* *<* *WF 1, WF 2*	*1.9″* *>* *1.25″*	*WF 3* *<* *WF 1, WF 2*	*1.9″* *>* *1.25″*
JUG flow	*WF 3* *<* *WF 1, WF 2*	ns	*WF 3* *<* *WF 1, WF 2*	ns
RENA flow	*WF 1* *>* *WF 3*	ns	*WF 1* *>* *WF 3*	ns
RENV flow	*WF 1* *>* *WF 3;* WF1 > WF 2	*1.9″* *>* *1.25″*	*WF 1* *>* *WF 3*	*1.9″* *>* *1.25″*

Italics text represents statistical significant (p < 0.05), normal text represents a statistical trend (p < 0.1), and non-significant differences are represented by ns

**Table 3:** Statistical dependence results from multivariate analysis that include possible interactions between chest compression release time and chest compression depth

Measurement	Model 4: WF + Depth + WF*Depth analysis		
	WF	Depth	WF*depth
AOP	*WF 2* *>* *WF 1, WF 3*	1.9″ > 1.25″	WF 2 > WF 1, WF 3 at 1.9″
RAP	WF 2 > WF 1	*1.25″* *>* *1.9″*	WF 1 < WF 2, WF 3 at 1.9″
CPP	*WF 2* *>* *WF 1, WF 3;* WF 1 > WF 3	*1.9″* *>* *1.25″*	ns
EtCO_2_	*WF 1* *>* *WF 2, WF 3*	1.9″ > 1.25″	*WF 1* *>* *WF 2, WF 3 at 1.9″*
AOR flow	ns	ns	WF 3 < WF 2 at 1.25″
IVC flow	WF 3 < WF 1	*1.9″* *>* *1.25″*	*WF 1* *>* *WF 2, WF 3 at 1.9″; WF 2* *>* *WF 1 at 1.25″*
CAR flow	*WF 3* *<* *WF1, WF 2*	*1.9″* *>* *1.25″*	*WF 3* *<* *WF 1,WF 2 at 1.9″; WF 3* *<* *WF 1 at 1.25″*
JUG flow	*WF 3* *<* *WF 1, WF 2*	ns	*WF 3* *<* *WF 1, WF 2 at 1.9″*
RENA flow	*WF 1* *>* *WF 3*	ns	*WF 3* *<* *WF 1, WF 2 at 1.9″*
RENV flow	*WF 1* *>* *WF 2, WF 3*	1.9″ > 1.25″	*WF 1* *>* *WF 2 at 1.9″; WF 3* *<* *WF 1, WF 2 at 1.25″*

Italics text represents statistical significant (p < 0.05), normal text represents a statistical trend (p < 0.1), and non-significant differences are represented by ns

A random intercept (for each subject) was included in the linear model to accommodate the cross-over repeated measures design of the experiment. The resulting correlation matrix of the residuals from a single subject is a compound symmetry matrix, meaning that all residuals from a subject are equally correlated with each other. Residuals from different subjects are considered uncorrelated. Residuals are assumed to be normally distributed. This kind of linear model is known as a linear mixed model (LMM).

We used Type III Tests of fixed effects to guide us in determining which covariates were significantly associated with each of the response variables. The *p* value for this test is computed as the tail area beyond a statistic test from an F-distribution, where the denominator degrees of freedom is computed accordingly to the Satterthwaite approximation method.

If a covariate was found significant, least square means for the response were computed for each level of the covariate and multiple comparisons (among the levels of the covariate) were performed using *t* test. The p-values calculations were adjusted for multiple comparisons using the simulate method in SAS. The conclusions of our results were reached by a step-wise approach that involved determining fixed effect significance in the univariate models first and then confirming such significance in the multivariate models.

The linear mixed models fitting as well as the statistical tests were obtained using PROC GLIMMIX on SAS version 9.3.

## Results

### Abdominal aortic blood flow

**Fig. 2:**
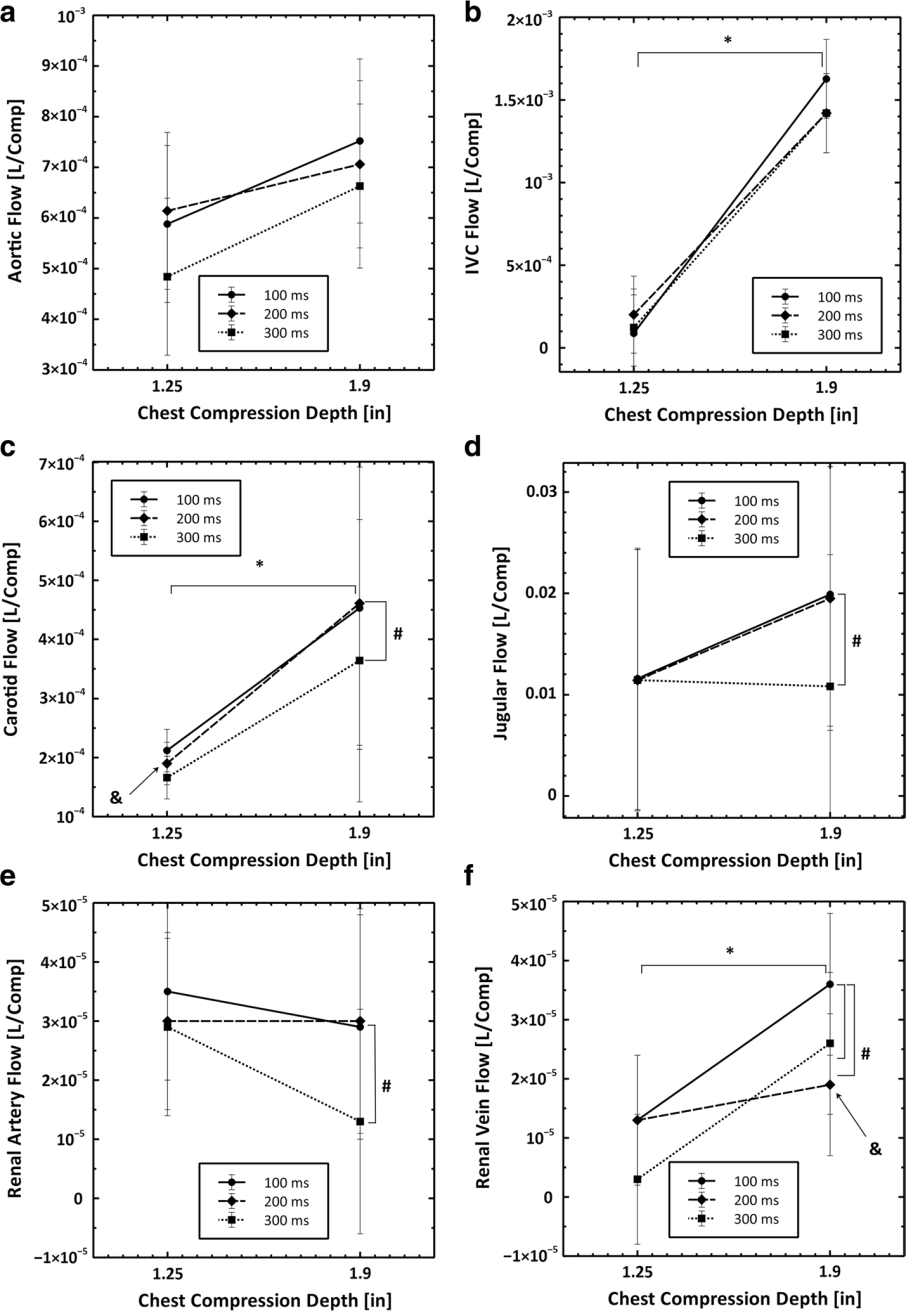
Plots showing the interaction between chest compression depth and chest compression waveform on the six blood flows that were measured in these experiments. All data are reported as liters per compression. The *number sign* symbol indicates statistical significance (p < 0.05) between waveforms. The *asterisk* symbol indicates statistical significance (p < 0.05) between chest compression depths. The *ampersand* symbol indicates statistical significance (p < 0.05) for interactions between the chest compression depth and the compression release time. **a** Aortic flow, **b** IVC flow, **c** carotid flow, **d** jugular flow, **e** renal artery flow and **f** renal vein flow

A 300 ms release time trended toward less aortic blood flow than a 200 ms release time, particularly when the chest compression depth was 1.25″ as shown in Fig. 2a. While this result was not significant, this trend was found in models 1, 3, and 4.

### Inferior vena cava blood flow

A chest compression depth of 1.9″ resulted in significantly more blood flow in the IVC than a chest compression depth of 1.25″ (*p* < 0.05) as shown in Fig. 2b. While model 4, Table 3, found a trend for a compression release time effect, and a significant compression release time—compression depth effect, model 1 and model 3, Table 2, show that there was no chest compression release time effect.

### Carotid artery blood flow

A 300 ms release time generated significantly less carotid blood flow than a 100 or 200 ms release time (*p* < 0.05). Carotid blood flow was significantly higher with a 1.9″ compression depth. Carotid blood flow increased significantly more for a 200 ms release time at 1.9″ compression depths than it did for 100 or 300 ms release times. These outcomes are supported by all statistical models and shown in Fig. 2c.

### Jugular vein blood flow

A 300 ms release time generated significantly less jugular blood flow than a 100 or 200 ms release time (*p* < 0.05) as shown in Fig. 2d. While model 4, Table 3, found that chest compressions with a 300 ms release time had a significant interaction with compression depth, model 2 and model 3, in Table 2, found no depth effect.

### Renal artery blood flow

A 300 ms release time generated significantly less renal artery blood flow than compressions with a 100 ms release time (*p* < 0.05) and renal artery blood flow was not dependent on chest compression depth as shown in Fig. 2e. While model 4, Table 3, found that chest compressions with a 300 ms release time had a significant interaction with chest compression depth, model 2 and model 3, Table 2, found no depth effect.

### Renal vein blood flow

A 200 or 300 ms release time generated significantly less renal vein blood flow than compressions with a 100 ms release time (*p* < 0.05). Renal vein blood flow was significantly greater with 1.9″ chest compressions. Renal vein blood flow increased significantly less for a 200 ms release time at 1.9″ compression depth than blood flow generated by compressions with 100 or 300 ms release times at 1.9″ depth. These conclusions are supported by all statistical models and shown in Fig. 2f.

### Aortic pressure (AOP)

**Fig. 3:**
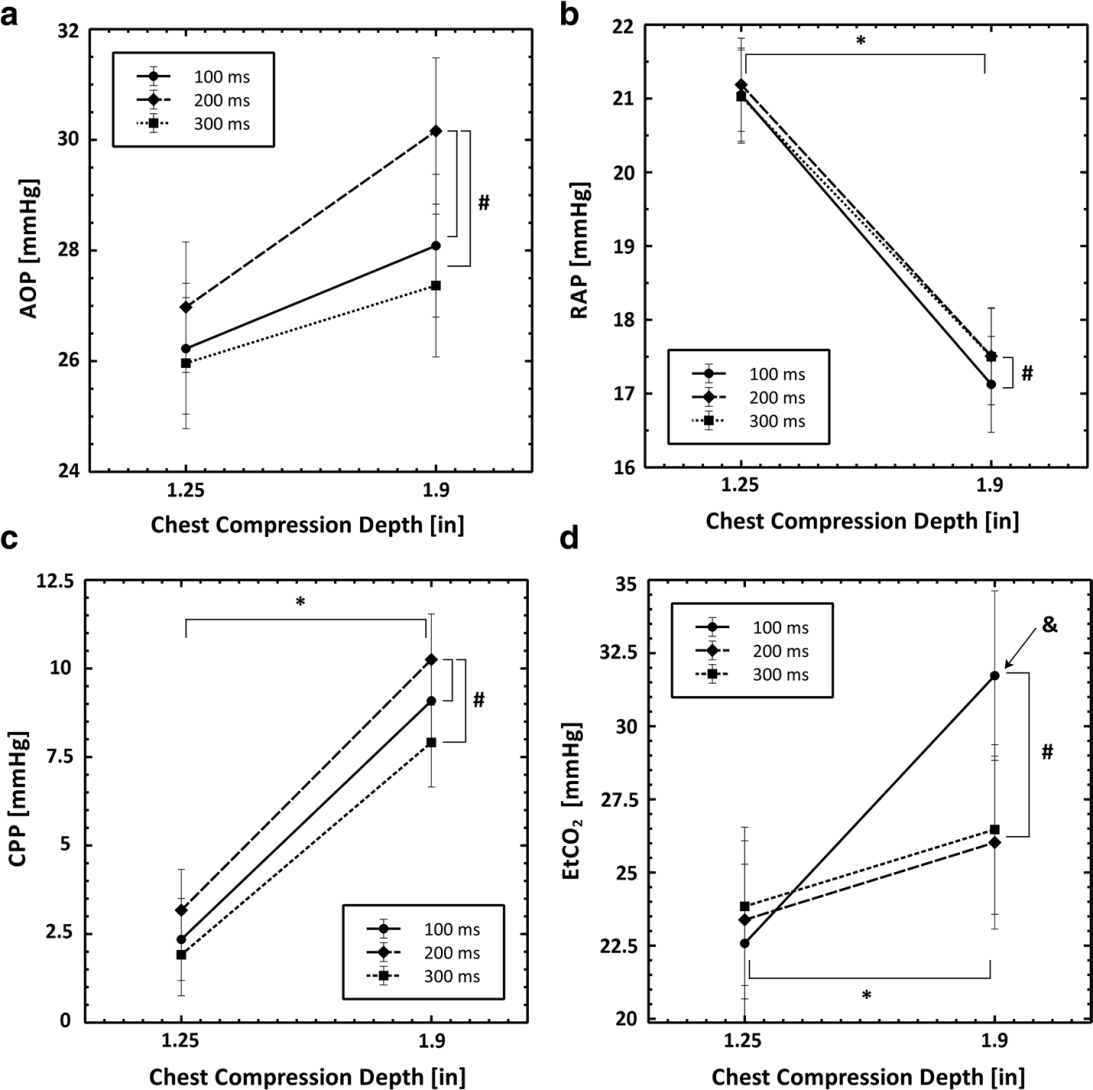
Plots showing the interaction between chest compression depth and chest compression waveform on blood pressures and EtCO_2_ that were measured in these experiments. All data are reported as mmHg. The *number sign* symbol indicates statistical significance (p < 0.05) between waveforms. The *ampersand* symbol indicates statistical significance (p < 0.05) between chest compression depths. The *ampersand* symbol indicates statistical significance (p < 0.05) for interactions between the chest compression depth and the compression release time. **a** Aortic pressure, **b** right atrial pressure, **c** coronary perfusion pressure and **d** end tidal carbon dioxide

A chest compression release time of 200 ms resulted in significantly higher AOP measurements than a 100 or 300 ms release time (*p* < 0.05) as shown in Fig. 3a. This result is confirmed by models 1, 3, and 4. Model 4 found a trend toward increased AOP measurements with a depth of 1.9″ and an interaction between chest compressions with a 200 ms release time with chest compression depth. However these trends were not confirmed in the univariate models.

### Right atrial pressure (RAP)

A chest compression release time of 200 ms resulted in significantly higher RAP measurements than a 100 ms release time (*p* < 0.05) as shown in Fig. 3b. A chest compression depth of 1.25″ resulted in a significantly higher RAP measurement than a 1.9″ compression depth. These results are supported by all four statistical models shown in Tables 2 and 3.

### Coronary perfusion pressure (CPP)

A chest compression release time of 200 ms resulted in significantly higher CPP measurements than a 100 ms release time or a 300 ms release time (*p* < 0.05) as shown in Fig. 3c. A chest compression depth of 1.9″ resulted in significantly higher CPP measurements than a 1.25″ release time. These results are supported by all four statistical models as shown in Tables 2 and 3.

### End-tidal carbon dioxide (EtCO_2_)

A chest compression release time of 100 ms resulted in significantly higher EtCO_2_ measurements than a 200 ms release time (*p* < 0.05) as shown in Fig. 3d. A chest compression depth of 1.9″ resulted in significantly higher EtCO_2_ measurements than a depth of 1.25″. There was a significant interaction between chest compression release time of 100 ms and chest compression depth. These results are supported by the results of models 1, 2, 3, and 4 as shown in Tables 2 and 3.

## Discussion

This is the first report describing the use of multiple extra-vascular flow probes to monitor chest compression generated blood flow. The six blood flows measured here provide a holistic, high fidelity window through which to study chest compression generated blood flow on a compression by compression basis. As described in the methods, we took a conservative approach to our statistical analysis. Conclusions about the impact of a change in chest compression release time or depth on blood flow were made based on the results of the four statistical models and required consistent results throughout all models. It is important to remember that chest compression generated blood flow oscillates, with periods of positive (physiologically normal direction) and negative (physiologically backward direction) blood flow within each compression, as shown in Additional file 2: Figure S2. The values reported in this paper are net forward flows per chest compression.

### Effect of chest compression release time

For most blood flows, a 300 ms release time resulted in significantly less blood flow than a 100 or 200 ms release time, as shown in Tables 2, 4, and Fig. 2. For example, carotid blood flow decreased ~20 % and jugular blood flow decreased ~45 % with a 300 ms release time and 1.9″ depth relative to the other release times. Aortic blood flow and IVC blood flow were also reduced by a 300 ms release time. Despite all chest compressions in this study conforming to the 2005 [5] or the 2010 [6] resuscitation guidelines, we observed that a 300 ms release time was detrimental to MCC-generated blood flow, regardless of chest compression depth. These results indicate that chest compression release time effects MCC generated blood flow, and is therefore an important metric of chest compression quality.

**Table 4:** Physiological outcomes reported by depth and release time from statistical model 4

Measurement	1.25″			1.9″ Depth			*p* *value*	
	100 ms	200 ms	300 ms	100 ms	200 ms	300 ms	WF (overall)	Depth
AOP (mmHg)	26.2 ± 1.18	27.0 ± 1.18	26.0 ± 1.18	28.1 ± 1.3	30.16 ± 1.32	27.27 ± 1.3	<*0.001*	0.0916
RAP (mmHg)	21.05 ± 0.63	21.19 ± 0.63	21.03 ± 0.63	17.12 ± 0.7	17.51 ± 0.66	17.5 ± 0.65	0.0650	<*0.001*
CPP (mmHg)	2.35 ± 1.16	3.17 ± 1.16	1.92 ± 1.16	9.09 ± 1.26	10.25 ± 1.3	7.91 ± 1.26	<*0.001*	<*0.001*
EtCO_2_ (mmHg)	22.58 ± 2.71	23.385 ± 2.7	23.84 ± 2.7	31.73 ± 2.9	26.02 ± 3.0	26.47 ± 2.9	*0.0045*	0.0615
AOR flow (L/comp)*10^4^	5.88 ± 1.55	6.14 ± 1.55	4.84 ± 1.55	7.52 ± 1.62	7.06 ± 1.65	6.63 ± 1.62	0.057	0.309
IVC flow (L/comp)*10^4^	0.88 ± 2.33	2.01 ± 2.33	1.23 ± 2.33	16.27 ± 2.39	14.20 ± 2.40	14.20 ± 2.39	0.067	<*0.001*
CAR flow (L/comp)*10^4^	2.12 ± 0.36	1.90 ± 0.36	1.66 ± 0.36	4.53 ± 0.39	4.61 ± 0.40	3.64 ± 0.39	<*0.001*	<*0.001*
JUG flow (L/comp)*10^4^	115.7 ± 129.0	114.3 ± 129.0	114.5 ± 129.0	198.9 ± 129.9	194.9 ± 130.2	108.3 ± 129.9	<*0.001*	0.134
RENA flow (L/comp)*10^4^	0.35 ± 0.15	0.30 ± 0.15	0.29 ± 0.15	0.29 ± 0.19	0.30 ± 0.19	0.13 ± 0.19	*0.003*	0.766
RENV flow (L/comp)*10^4^	0.13 ± 0.11	0.13 ± 0.11	0.03 ± 0.11	0.36 ± 0.12	0.19 ± 0.12	0.26 ± 0.12	*0.003*	0.079

All flows are reported as mean ± standard error. *p* values less than 0.05 are in italics

* Q × 10^4^ indicates that all flows (Q) have been multiplied by 10^4^, resulting in units of [100 μL/Comp]. To calculate units of [L/Comp] multiply reported values by 10^−4^

Physiological measures of chest compression efficacy followed a different pattern. As shown in Table 2 and Fig. 3, a chest compression release time of 200 ms optimized aortic pressures. A release time of 100 ms optimized right atrial pressure and EtCO_2_. The CPP is defined as the difference between the aortic pressure and right atrial pressure during decompression. As shown in Table 2 and Fig. 3c, the CPP was optimized by chest compressions with a 200 ms release time.

### Effect of chest compression depth

While fewer measured parameters were dependent on chest compression depth, those that were depth dependent uniformly favored deeper compressions. Right atrial pressure was decreased with a MCC depth of 1.9″ inches, as shown in Table 2 and Fig. 3, resulting in a significant improvement in CPP. EtCO_2_ was significantly increased with deeper chest compressions. Net forward blood flow was increased in the IVC, the carotid artery, and the right renal artery with 1.9″ deep chest compressions relative to 1.25″ deep chest compressions. Surprisingly, aortic pressure, aortic blood flow, jugular vein blood flow and renal artery blood flow were not affected by changes in chest compression depth.

### Examining the depth effect on anatomically paired flows

Comparing the effect of changes in chest compression on blood flows in anatomically matched arteries and veins highlights the following contradictory results: IVC blood flow increases significantly with a depth of 1.9″ while aortic blood flow does not; carotid blood flow increases significantly with a depth of 1.9″ while jugular blood flow does not; renal vein blood flow increases significantly with a depth of 1.9″ while renal artery blood flow does not. Blood flow probes were co-located. In the neck, the right common carotid artery and right external jugular vein were instrumented. The right renal artery and vein were instrumented. The abdominal aorta and IVC were instrumented on the immediate thoracic side of the renal branches of the abdominal aorta and inferior vena cava. While it would be naive to expect the blood flows in the respective vessels to match exactly on a volumetric flow rate basis; it is unexpected that an increase in blood flow in one vessel of an anatomically matched set is not shared by the other. These data suggest that deeper chest compressions change the path the blood takes through the vasculature in addition to changing the total amount of forward blood flow. This raises the possibility that deeper chest compressions are better because they increase flow to the critical tissues, such as the brain, as opposed to increasing forward flow globally.

These findings fall outside of the predictions of the cardiac pump model [1], the thoracic pump model [2–4], or any of the numerical models that have been published to date [21–23]. One possible explanation for the difference between the prediction of the models and our experimental results is that the anatomical complexity of the non-thoracic vasculature is not included in the numerical models. Models that include more complicated representations of the non-thoracic vasculature may provide more insight into the connection between chest compression depth and the vascular path the blood follows.

### Interactions between MCC release time and MCC depth

Several physiological measures exhibited significant interactions between chest compression depth and chest compression release time. Blood flow generated by MCC with a 200 ms release time in the carotid artery increased significantly more than MCC with a 100 or 300 ms release time when compression depth was increased from 1.25″–1.9″, as shown in Table 3 and Fig. 2c. Blood flow in the renal vein exhibited the same behavior. In addition, the EtCO_2_ values generated by MCC with a 100 ms release time increased significantly more than MCC with a 200 or 300 ms release time when compression depth was increased from 1.25–1.9, as shown in Table 3 and Fig. 3d. The causes of these interactions are not clear. Dimensional analysis of time and depth suggest that release velocity (depth/time) or release power (Force*depth/time) could be related to these findings.

### Chest compression release affects CPR quality

There is a growing interest in chest compression release in the resuscitation literature. To date, chest compression release has been discussed in terms of incomplete chest compression release, referred to as leaning [18], or chest compression release velocity [10, 11]. While leaning has been shown to be common during resuscitations [17, 18], the evidence that leaning is detrimental to outcomes is primarily from animal research without the benefit of real time systemic blood flow information [19, 20]. While our data on the effect of depth or release time can provide insight into possible kinematic effects of incomplete chest compression release, our study was not designed to address this question directly.

Our results are more readily compared to the studies of chest compression release velocity [10, 11]. The paper by Cheskes et al. found that release velocity was not predictive of survival in multivariate models adjusted for Utstein measures, and the paper by Kovacs et al. found a survival benefit associated with faster release velocities. In these papers, chest compressions were delivered manually without active decompression and release velocity was estimated, without a detailed description of the method, by integrating the acceleration signal captured by CPR quality measuring defibrillators. Differences in release velocity measurement method and chest compression delivery complicate comparison of the findings in these studies with the results of the present study.

The data reported here provides important quantitative insight into how sternum release kinematics can impact blood flow. Our data in combination with the clinical data on release velocity suggests that chest compression release can have a significant impact on chest compression generated blood flow, beyond the effect of incomplete release. However, there is not good agreement about which velocities matter. Release velocity has been treated as a categorical variable with the following bins: v < 300 mm/s, 300 mm/s ≤ v < 400 mm/s, v ≥ 400 mm/s. Kovacs et al. found significant survival differences between these release velocities [11]. In our study, only one compression waveform (1.9″ deep with 100 ms release time) had a release velocity greater than 400 mm/s, and only one compression waveform (1.25″ deep with 100 ms release time) had a release velocity between 300 and 400 mm/s. All other waveforms had release velocities slower than 300 mm/s. Our finding, as shown in Tables 2 and 3, is that a 300 ms release time was detrimental to blood flow. We did not find that chest compressions with a 100 ms release were different than chest compressions with a 200 ms release, which is what the results of Kovacs et al. would predict.

We believe this discrepancy is significant and requires further study. It is possible that the survival benefit described by Kovacs et al., was not conferred by improved blood flow, but instead through some other mechanism that is dependent on chest compression kinematics. Our finding that EtCO_2_ was highest with a 100 ms release time suggests that gas exchange in the lungs is a possible explanation. Another possibility is that release velocity or release time is not the kinematic factor that dominates blood flow. It is interesting to note that two of our chest compression waveforms had nearly identical release velocities: 1.9″ deep with a 300 ms release time, v = 160 mm/s; 1.25″ deep with a 200 ms release time, v = 158.79 mm/s, yet our statistical finding is that the 300 ms release time generates less blood flow than the 200 ms release time. This suggests that it may be the release pause, defined as time at full chest recoil between compressions, which is the dominating factor; not release velocity or release time. As this was not studied in the clinical papers, we cannot compare our release pause times to their results. Finally, this difference may represent the affect of active decompression on CPR hemodynamics. The MCC delivered in this study guaranteed that the sternum reached its initial position in each chest compression. This would not be true during manual chest compressions without active decompression. Therefore, during manual chest compressions, release velocities slower than 400 mm/s may be a symptom of detrimental changes in thoracic mechanics which are reducing the efficacy of CPR. This possibility highlights the importance of a kinematic understanding of chest compression generated blood flow as well as the importance of getting more accurate measurements during clinical resuscitations.

### Study limitations

This study was performed on adolescent swine. While there are clear physiological differences between the animal subjects used in this study and the typical cardiac arrest patient, the chosen size/weight and species are the most commonly used in pre-clinical cardiac arrest/CPR studies.

The effects of positive pressure ventilation are not reported. It is expected that increases in intra-thoracic pressure due to positive pressure ventilation will alter MCC hemodynamics. The design of the experiments allowed for any potential confounding effect of positive pressure ventilation to be spread evenly among the different depths and release times tested.

The sternum was coupled to the chest compressor using self adhesive hook and loop fasteners to enable positional control of the sternum. As a result, the decompression phase of the MCC was active, as found in some commercially available mechanical chest compression devices. The relationships between MCC depth and release times reported here may not be generalizable to chest compressions that do not include active decompression.

Chest compressions are surprisingly complex mechanical oscillations. Changing the release time, as we did here, concomitantly changes the release hold time and the chest compression duty cycle. At this point it is not clear which of these changes is responsible for the differences in blood flows observed in this study. However, this does not detract from the finding that slower release impairs chest compression generated blood flow.

We have not reported data that would enable the interpretation of these blood flow measurements through the lens of the cardiac pump or thoracic pump models. This was done in part to allow a discussion of the results without the constraints or assumptions that underpin any modeling effort; particularly because there are aspects of chest compression generated blood flow that remain controversial. We hope that these data can be used in the future to inform our mechanical understanding of chest compression generated blood flow.

Animals in this study were never resuscitated. As a result, the effects of changes in chest compression depth or release time on return of spontaneous circulation and survival remain unknown.

The surgical procedures were invasive and the placement of extra-vascular flow probes may induce vascular responses that affect blood flow.

## Conclusions

We have developed a high fidelity swine model to measure six blood flows during cardiac arrest. In future experiments, this model may be a useful platform from which to study questions related to the impact of time on chest compression generated blood flow. This is also a promising model to address remaining controversies related to the cardiac pump and thoracic pump models for chest compression generated blood flow.

In conclusion, 1.9″ chest compressions with moderate release times (100–200 ms) generated the best blood flows in this swine model of CPR. A 300 ms release time interfered with chest compression generated blood flow. A 200 ms release time at a depth of 1.9″ optimized blood pressures, particularly the CPP. A 100 ms release time at a depth of 1.9″ optimized EtCO_2_. Chest compression release time is an important metric of CPR quality. Further study is required to understand how to best optimize hemodynamics over varying periods of CPR and how those optimization strategies relate to outcomes.

## Additional files

10.1186/s12938-015-0095-4 Schematic of the chest compression parameters controlled to generate the 6 distinct waveforms tested in these experiments. The two parameters that were changed were #1: Depth and #4, release time.
10.1186/s12938-015-0095-4 This figure shows the six measured blood flows approximately 10 min after the initiation of CPR. Several aspects of the flow should be noted. First, there is a transition between waveforms at ~1632 s. Second, all flow are traveling in the physiologically normal (positive) direction and the physiologically abnormal (negative) direction. These data confirm that CPR generates oscillatory flow as opposed to directional flow.
