# Supplementary figures and images for: Developing a kinematic understanding of chest compressions: the impact of depth and release time on blood flow during cardiopulmonary resuscitation
Source: Biomed Eng Online. 2015 Nov 4;14:102. doi: 10.1186/s12938-015-0095-4 (PMC4634731; doi:10.1186/s12938-015-0095-4)

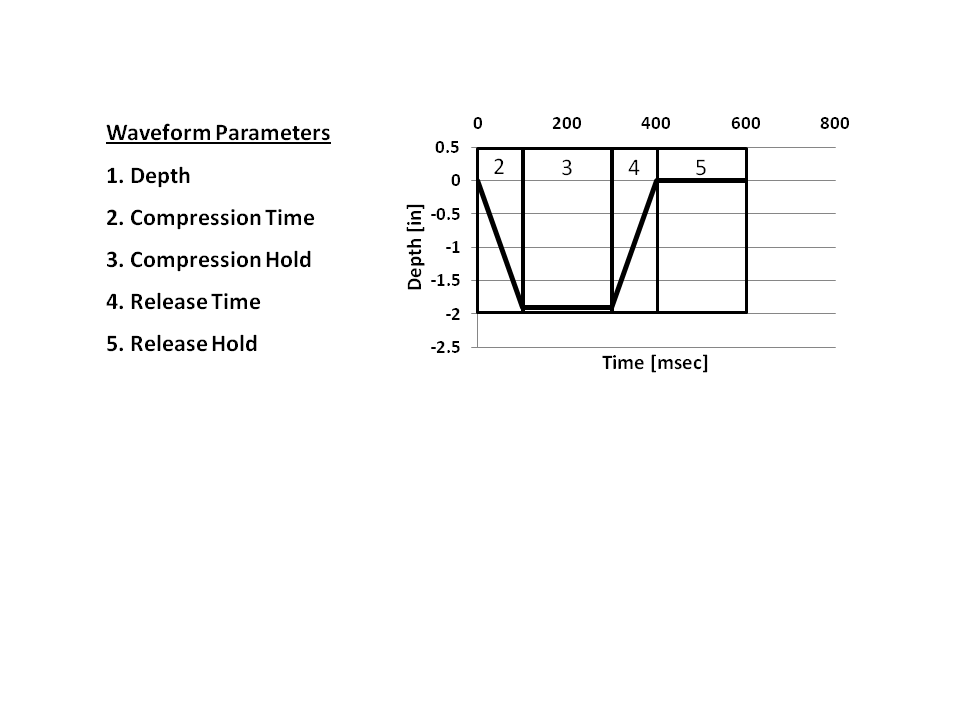

Supplement: Supplementary file 1 — 10.1186/s12938-015-0095-4 Schematic of the chest compression parameters controlled to generate the 6 distinct waveforms tested in these experiments. The two parameters that were changed were #1: Depth and #4, release time. [file 12938_2015_95_MOESM1_ESM.tif]

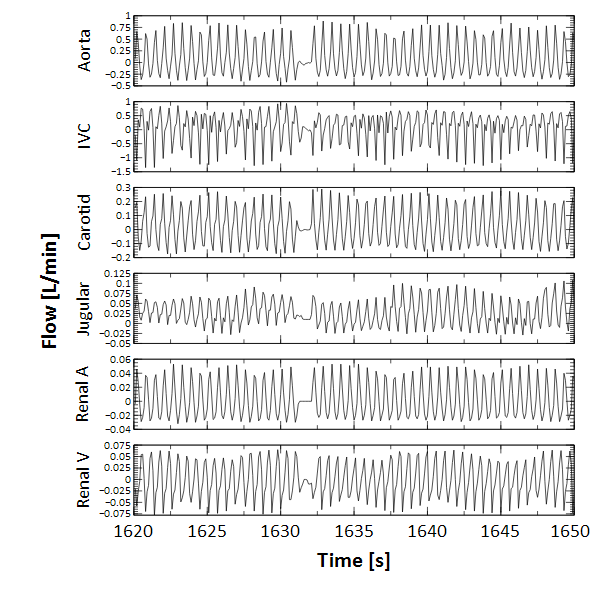

Supplement: Supplementary file 2 — 10.1186/s12938-015-0095-4 This figure shows the six measured blood flows approximately 10 min after the initiation of CPR. Several aspects of the flow should be noted. First, there is a transition between waveforms at ~1632 s. Second, all flow are traveling in the physiologically normal (positive) direction and the physiologically abnormal (negative) direction. These data confirm that CPR generates oscillatory flow as opposed to directional flow. [file 12938_2015_95_MOESM2_ESM.tiff]
